# Phenotypic and molecular phylogeny of *Klebsiella pneumoniae* isolated from respiratory-diseased pet cats in Iraq

**DOI:** 10.5455/javar.2025.l926

**Published:** 2025-06-02

**Authors:** Ahlam A. S. Al-Galebi, Mithal K. A. Al-Hassani, Hadaf Mahdi Kadhim, Hasanain A. J. Gharban

**Affiliations:** 1 Department of Biology, College of Education, University of Al-Qadisiyah, Al-Diwaniyah, Iraq; 2 Department of Internal and Preventive Veterinary Medicine, College of Veterinary Medicine, University of Wasit, Wasit, Iraq

**Keywords:** *16S rRNA gene*, antibiotic sensitivity, feline respiratory diseases, nosocomial pathogens, NCBI, sequencing data

## Abstract

**Objective:**

Investigation of *Klebsiella pneumoniae* in respiratory-diseased pet cats, estimation of antibiotic sensitivity, and molecular phylogeny of local *K*. *pneumoniae* to identify its identity to global isolates.

**Methods:**

Totally, 127 feline cases with various respiratory signs were selected for the collection of the nasal swabs that were cultured to isolate *K*. *pneumoniae* and detect the antibiotic sensitivity. Further molecular phylogeny of positive *K*. *pneumoniae* isolates was done.

**Results:**

Findings of culture media and biochemical tests showed that 26.77% of nasal swabs were positive samples for *K*. *pneumoniae*. The screening for the antibiotic susceptibility reported a higher sensitivity to ceftiofur, ciprofloxacin, cefepime, amikacin, gentamicin, cefotaxime, and meropenem, as well as ceftazidime, ceftriaxone, and doxycycline, imipenem, as well as clotrimazole and tetracycline. In contrast, the more significant resistant *K*. *pneumoniae* isolates were detected to clarithromycin, clindamycin, amoxicillin, cefixime, chloramphenicol, erythromycin, cephalexin, cefadroxil, azithromycin, and nalidixic acid, whereas, significant semi-sensitivity was shown to tylosin. Molecular testing by polymerase chain reaction demonstrated that all isolates were *K*. *pneumoniae*. The genetics-based analysis of local *K*. *pneumoniae* isolates recorded an overall similarity (95.47%–100%) and changes/mutations (0.0004%–0.0084%), in particular to the National Center for Biotechnology Information-Iraqi isolate (Lc732203.1).

**Conclusion:**

This study indicates the high prevalence of *K*. *pneumoniae* in respiratory-diseased cats with significant appearance of antibiotic resistance in study isolates. Sequencing data referred to the close related association of study isolates to human *K*. *pneumoniae* isolates, suggesting the increased prevalence of nosocomial infections in veterinary medicine.

## Introduction

*Klebsiella pneumoniae* is one of the most widespread Gram-negative bacteria, which is closely related to the family of Enterobacteriaceae that additionally involves other known pathogens such as *Salmonella* spp. and *Escherichia coli* [1,2]. Although *K*. *pneumoniae* is a normal microbiota, it represents an important nosocomial opportunistic bacterium due to the presence of various virulence factors, including a capsule, antigens, fimbrial adhesion, an iron acquisition system, and the formation of a biofilm that allows it to evade the innate immunity of a host [3–5]. Typically, lipopolysaccharide (O) and capsular (K) antigens are the main factors on the cell surface, which contribute to the pathogenicity and classification of the bacterium into various serotypes [6]. In the last decade, multidrug resistance has increased significantly due to biofilm formation, efflux pumps, the production of β-lactamases, and additional enhancement due to the modification of enzymes as well as the loss of porin [7]. Incidence of resistance causes a reduction in the effectiveness of traditional drugs applied commonly to treat *K*. *pneumoniae* and increases the rates of morbidity and mortality [8].

In cats, respiratory infections occur frequently in high-density localities such as feral cat colonies, catteries, shelters, and breeding due to various viral, bacterial, fungal, and protozoal pathogens that negatively impact feline health and cause different symptoms such as nasal discharge, cough, sneezing, lethargy, loss of appetite, and trouble breathing [9–11]. To prevent a worsening of infection, antibiotics are used largely; however, the selection drug of choice is still needed, especially in severe systemic diseases that have a much less favorable prognosis [12,13]. Therefore, continuous surveillance and monitoring of respiratory bacterial infections and their resistances to antibiotics will not only guide the veterinarians throughout the control of infections but also support the investigation of respiratory diseases [14,15].

Due to the severity of *K*. *pneumoniae* infection in both humans and animals with the absence of such studies in Iraq, we investigate *K*. *pneumoniae* in respiratory-diseased pet cats and estimate the antibiotic sensitivity. Molecular phylogeny of local *K*. *pneumoniae* was done to identify its identity to global isolates.

## Materials and Methods

### Ethical approval

The Scientific Committee of the Department of Biology (College of Education, University of Al-Qadisiyah) licensed the current study (Approval No. 122/QU-EC/18-10-2023).

### Preparation of culture media and broth

According to manufacturers’ instructions, Brain Heart Infusion (BHI) broth (HiMedia, India) and the media of Blood and MacConkey Agars (HiMedia, India) were prepared to transport swab samples and *K*. *pneumoniae* isolate.

### Samples

Totally, 127 feline cases with various respiratory signs were selected during October (2023)–February (2024) from different private veterinary clinics in Baghdad province (Iraq). Under aseptic conditions, nasal swab samples were collected, kept in plastic tubes containing the BHI broth, and transported cooled to the laboratory.

### Isolation

The streaking method of nasal swabs was used to isolate *K*. *pneumoniae* on plates of blood and MacConkey agars. After incubation (37°C/24 h), the suspected colonies that were distinguished based on their morphological characteristics were transferred to each slant for more purification [16].

### Gram staining

Thin smears were made from the pure colonies on a glass slide, safranin-stained, and observed under oil magnification of light microscopy (MEIJI, Japan) at 1,000x.

### Biochemical testing

After purification, the colonies of suspected *K*. *pneumoniae* isolates were tested biochemically using the Voges-Proskauer, urease, triple sugar iron agar (TSI), oxidase, methyl red, H2S, gelatin hydrolysis, and gas production [16,17].

### Antibiotic sensitivity testing

After isolation and purification, *K*. *pneumoniae* isolates were examined by the Kirby–Bauer test to detect their sensitivity and resistance to 24 antibiotics (Roseto degli Abruzzi, Italy), including amikacin, amoxicillin, azithromycin, cefadroxil, cefepime, cefixime, cefotaxime, ceftazidime, ceftiofur, ceftriaxone, cephalexin, chloramphenicol, ciprofloxacin, clarithromycin, clindamycin, clotrimazole, doxycycline, erythromycin, gentamicin, imipenem, meropenem, nalidixic acid, tetracycline, and tylosin.

### Molecular testing

Following the manufacturers’ instructions for the Presto^™^ Mini genomic deoxyribonucleic acid Bacteria Kit (Geneaid, Taiwan), DNAs were extracted from the colonies of *K*. *pneumoniae* samples and then examined by the Nanodrop spectrophotometer system. Targeting the *16S rRNA* gene, asterMix tubes were prepared at 50 μl using the AccuPower^®^ Taq PreMix Kit (Bioneer, South Korea) and designing one set of primers [(F: 5’CTC GAA ACG CAT GAG CAT GG -3’) and (R: 5’GCG ACA GTG CGT TAT CGT TC-3’)] according to the National Center For Biotechnology Information (NCBI)-GenBank *K*. *pneumoniae* isolate (NZ_ JAYMFY010000085.1). DNA amplification in the Thermal Cycler system (Thermo Scientific, USA) was done as described [18], and positives were identified at 787 bp.

### Genotyping

Five positive DNA samples were sequenced, submitted to the NCBI database, and subjected to phylogenetic analysis using the MEGA-11 software.

### Statistical analysis

In the GraphPad Prism software (version 9.4.1), one-way ANOVA and *t*-test were performed to find out significant differences in antibiotic sensitivity and between the isolation and polymerase chain reaction (PCR) assay results. Study values were seen as significantly different when *p* < 0.05 [18].

## Results

### Morphological and biochemical characterizations

**Figure 1.**
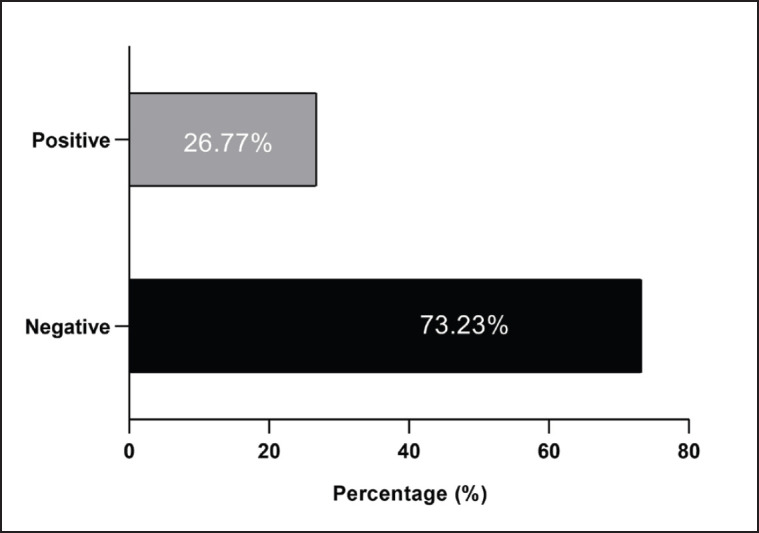
Total results for detection of *Klebsiella pneumoniae* isolates by culture and biochemical tests among totally 127 feline swab samples.

Findings of culture media and biochemical tests showed that 26.77% (34/127) of nasal swabs were positive samples to *K*. *pneumoniae* as shown in Figure 1. On blood agar, K. pneumoniae appeared as mucoid, opaque, smoothly raised colonies, while on MacConkey agar, they were considered mucoid, pink to red colored, large, circular, and convexly elevated colonies. Microscopically, K. pneumoniae was observed as a large, Gram-negative, rod-shaped bacterium. Concerning the biochemical tests, suspected *K*. *pneumoniae* isolates were positive to Voges proskauer, urease, and gas production; acid/acid reaction in TSI; and negatives to H2S production, gelatin hydrolysis, methyl red, and oxidase.

### Antibiotic susceptibility

The findings of screening the susceptibility of bacterial isolates toward different 24 antibiotics reported a significantly higher sensitivity (*p* ≤ 0.0001) to ceftiofur (97.06%), ciprofloxacin (82.35%), cefepime (79.41%), amikacin (76.47%), gentamicin (73.53%), cefotaxime and meropenem (67.65%), ceftazidime, ceftriaxone, doxycycline (61.76%), and imipenem (55.88%), as well as clotrimazole and tetracycline (44.12%). In contrast, the more significant (*p* ≤ 0.0001) resistant *K*. *pneumoniae* isolates were detected to clarithromycin (100%), clindamycin (97.06%), amoxicillin, cefixime, and chloramphenicol (85.29%), erythromycin (70.59%), cephalexin (67.65%), cefadroxil (64.71%), azithromycin (50%), and nalidixic acid (47.06%), whereas, significant semi-sensitivity (*p* ≤ 0.0001) was shown to tylosin (52.94%) as shown in Table 1.

**Table 1.** Antibiotic susceptibility of *Klebsiella pneumoniaw* by disc diffusion test (total no: 34).

Antibiotic	Dose (μg)	Antibiotic susceptibility		
		Resistant	Semi-sensitive	Sensitive
Amikacin	30	8 (23.53%)	0 (0%)	26 (76.47%)
Amoxicillin	10	29 (85.29%)	4 (11.77%)	1 (2.94%)
Azithromycin	15	17 (50%)	11 (32.35%)	6 (17.65%)
Cefadroxil	30	22 (64.71%)	8 (23.53%)	4 (11.77%)
Cefepim	10	5 (14.71%)	2 (5.88%)	27 (79.41%)
Cefixime	5	29 (85.29%)	5 (14.71%)	0 (0%)
Cefotaxime	30	8 (23.53%)	3 (8.82%)	23 (67.65%)
Ceftazidime	10	13 (38.24%)	0 (0%)	21 (61.76%)
Ceftiofur	30	1 (2.94%)	0 (0%)	33 (97.06%)
Ceftriaxone	30	8 (23.53%)	5 (14.71%)	21 (61.76%)
Cephalexin	30	23 (67.65%)	9 (26.47%)	2 (5.88%)
Chloramphenicol	10	29 (85.29%)	5 (14.71%)	0 (0%)
Ciprofloxacin	5	6 (17.65%)	0 (0%)	28 (82.35%)
Clarithromycin	15	34 (100%)	0 (0%)	0 (0%)
Clindamycin	10	33 (97.06%)	1 (2.94%)	0 (0%)
Cotrimazol	50	12 (35.29%)	7 (20.59%)	15 (44.12%)
Doxycycline	30	11 (32.35%)	2 (5.88%)	21 (61.76%)
Erythromycin	15	24 (70.59%)	8 (23.53%)	2 (5.88%)
Gentamicin	30	7 (20.59%)	2 (5.88%)	25 (73.53%)
Imipenem	10	11 (32.35%)	4 (11.77%)	19 (55.88%)
Meropenem	10	9 (26.47%)	2 (5.88%)	23 (67.65%)
Nalidixic acid	30	16 (47.06%)	6 (17.65%)	12 (35.29%)
Tetracycline	30	10 (29.41%)	9 (26.47%)	15 (44.12%)
Tylosin	30	11 (32.35%)	18 (52.94%)	5 (14.71%)
*p*-value		0.0001	0.0001	0.0001
CI		34.01–58.15	8.174–19.03	26.58–54.06

### Molecular and phylogenetic analysis

**Figure 2.**
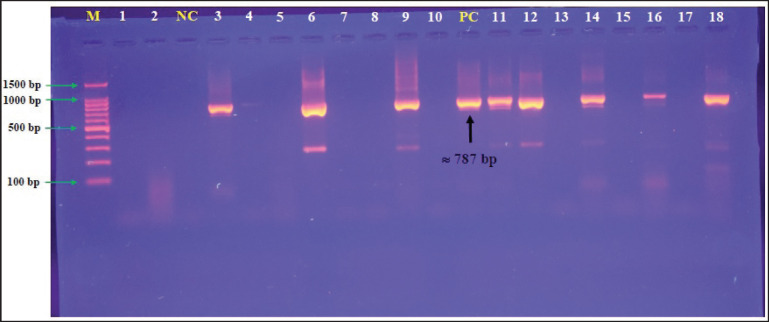
Electrophoresis of 1.5% agarose gel stained with ethidium bromide. Lane M: Ladder marker (100–1,500 bp); Lanes 1–5: Reprentative positive samples at 787 bp.

**Figure 3.**
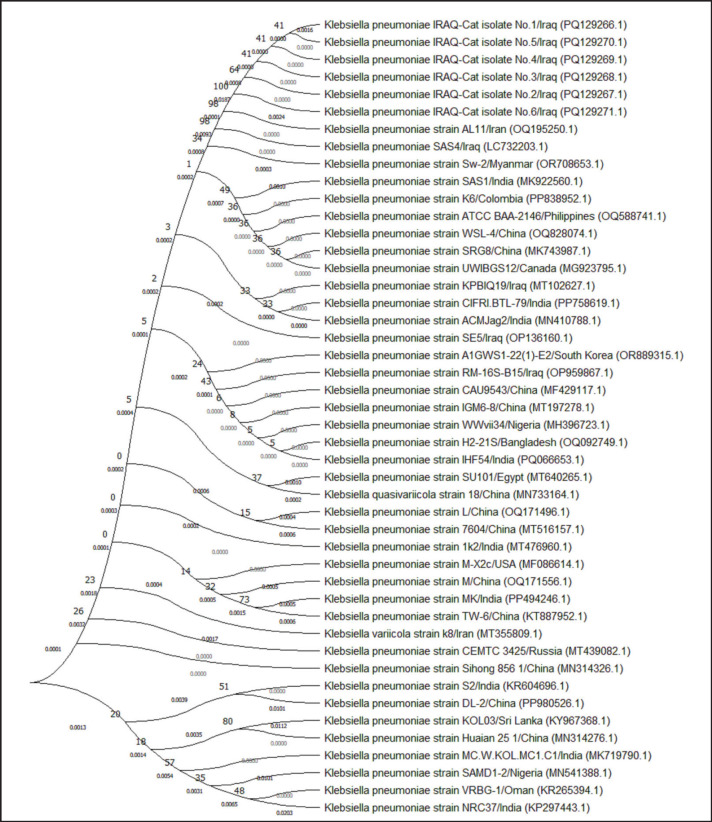
Phylogenetic tree analysis of local IRAQ-Cat *Klebsiella pneumoniae* isolates comparison with the NCBI-BLAST *K*. *pneumoniae* isolates/strains. The analysis was built using the Unweighted Pair Group technique with Arithmetic Mean (tree method), and the evolutionary distances were calculated using the Maximum Composite Likelihood method in MEGA 11.

Targeting the *16S rRNA* gene, a specific conventional PCR assay detected that all study samples (total no. 34) were positive *K*. *pneumoniae* isolates, as shown in Figure 2. Sequencing data of five positive K. pneumoniae isolates were received, named IRAQ-Cat isolate No.1, IRAQCat isolate No.2, IRAQ-Cat isolate No. 3, IRAQ-Cat isolate No.4, and IRAQ-Cat isolate No. 5, submitted in the NCBI database under specific access numbers (PQ129266.1, PQ129267.1, PQ129268.1, PQ129269.1, and PQ129270.1), and analyzed phylogenetically to explore nucleotide similarity (*) and mutations/changes with *K*. *pneumoniae* isolates/strains in NCBI-GenBank. The genetics-based analysis of local *K*. *pneumoniae* isolates recorded an overall similarity (95.47%–100%) and changes/mutations (0.0004%–0.0084%) in particular to the NCBI-Iraqi isolate (Lc732203.1) (Table 2, Fig. 3).

**Table 2.** Homology sequence identity (%) of local *Klebsiella pneumonia* isolates and the NCBI-BLAST *K*. *pneumonia* isolates.

Local isolate		NCBI-BLAST isolate			
Name	Access No.	Access No.	Country	Host	Identity (%)
IRAQ-Cat isolate No.1	PQ129266.1	LC732203.1	Iraq	Human	98.85
IRAQ-Cat isolate No.2	PQ129267.1	LC732203.1	Iraq	Human	99.17
IRAQ-Cat isolate No.3	PQ129268.1	LC732203.1	Iraq	Human	99.83
IRAQ-Cat isolate No.4	PQ129269.1	LC732203.1	Iraq	Human	99.83
IRAQ-Cat isolate No.5	PQ129270.1	LC732203.1	Iraq	Human	99.67

## Discussion

In the Enterobacteriaceae family, *Klebsiella* represents the second cause of nosocomial infections after *E. coli*, with noting that almost all *Klebsiella* diseases were attributed to *K*. *pneumoniae* [19]. In a variety of disorders in cats, the emergence of infectious upper and lower respiratory diseases caused by *K*. *pneumoniae* has shown a significant increase in the last decade due to the frequent occurrence of multidrug resistance [13,20]. However, in Iraq, *Mycoplasma* spp. is the only pathogen diagnosed in cats [21]. In this study, examination of an overall 127 nasal swabs using the traditional diagnostic methods (culture, biochemical tests, and Gram-staining) revealed 26.77% positive cats to *K*. *pneumoniae* infection. Comparatively, various studies reported that *K*. *pneumoniae* was seen in 3% of Germany’s cats [22], 14% in South Korea [23], 40% in Indonesia [24], and 2.4% in China [25]. In a previous retrospective analysis of infectious respiratory diseases in cats, Foster et al. [26] mentioned that *K*. *pneumoniae* represents one of the most common infectious bacterial causes in cats, especially feline pneumonia. However, Aslam et al. [20] concluded that infectious respiratory diseases in cats are complicated, particularly in unvaccinated kittens and young adults that live in households or shelters.

Identification of bacteria, not their sensitivity to antibiotics, has been what veterinary studies pay special attention to when sharing their results on microbes found in airway samples; valuable information could only guide the therapy when accompanied by susceptibility patterns [27,28]. Bacteria have, over overtime, developed resistance to antibiotics, and several multidrug-resistant microbes were initiated to guide more suitable choosing of drugs [29–31]. Resistant strains of *K*. *pneumoniae* are of great health importance because decreasing effect of different drugs [24]. Our findings reported that *K*. *pneumoniae* isolates have variable degrees of susceptibility to selected antibiotics. Possible causes of resistance are the exceptional capability to acquire exogenous hypervirulence-encoding and resistance-encoding genetic elements, make changes or mutations in the protein-coding genes, and produce β-lactamase enzymes [32–34].

In comparison with other researchers, *K*. *pneumoniae* isolates were reported with various findings among animals and humans. In cats, Epstein et al. [27] found that K. pneumoniae isolates were lessly to be susceptible to ticarcillin/clavulanate, enrofloxacin, chloramphenicol, amoxicillin/clavulanate, and ampicillin. De Sousa et al. [35] recorded that antimicrobial resistance genes found in *K*. *pneumoniae* coming from animals make them resistant to many drugs, proving that animals can carry important resistance factors. Li et al. [36] observed that respiratory tract infections in cats caused by *K*. *pneumoniae* presented a higher antimicrobial resistance, mainly for piperacillin, trimethoprim/sulfamethoxazole, cefotaxime, and cefuroxime. In one study, isolates of *K*. *pneumoniae* obtained from clinical cats in Bogor, Indonesia, were resistant to oxytetracycline, tetracycline, enrofloxacin, and gentamicin due to the presence of different genes such as *blaTEM*, *tetA*, *blaSHV*, *aac3-IV*, and *qnrS* at different rates of prevalence: 100%, 57.2%, 30.4%, 33.3%, and 28.5%, respectively [24]. Zhang et al. [25] showed that *K*. *pneumoniae* isolates have greater resistance to trimethoprim/sulfamethoxazole and amoxicillin/clavulanate.

Using certain molecular methods in microbiology, together with microbiologic tests, can result in effective and accurate findings [27,37]. Molecular-based PCR assays have recorded a high accuracy in the detection of *Klebsiella* species in comparison to other traditional diagnostic techniques [38,39]. In addition, high speed and low contamination levels make PCR an appealing method in the identification of various infectious pathogens [40]. In this study, all of the hospital-related bacteria turned out to be positive for *K*. *pneumoniae*, and this was confirmed by matching their DNAs. Consequently, by looking at the phylogenetic trees for some positive samples, we found that some of them were closely related to human types of *K*. *pneumoniae* stored in the NCBI and GenBank databases. Many researchers showed that using genetic tests was very accurate and could help identify *K*. *pneumoniae* bacteria, especially because the *16S*–*23S rRNA* genes found in these bacteria often stay similar but have enough differences to tell one type from another [41–43]. It has been shown that looking at the sequence of *16S*–*23S rRNA* genes makes it possible to tell different *Klebsiella* species and subspecies apart [42,44]. Marques et al. [45] found that the hospital-adapted *K*. *pneumoniae* in both cats and people shared 81.1% of their gene sequence and carried many genes that made these bacteria harmful or resistant to drugs.

This study has some limitations. First, the samples were not collected from all diseased dogs due to disagreement of their owners. Second, the dependence of the study on culture in screening might have resulted in missing some isolates. Third, only one gene was targeted in the confirmation of *K*. *pneumoniae* isolates, and this caused a missing in information about the strains of study isolates and their pathogenicity. Fourth, if a large number of samples are collected and tested using the traditional and molecular phylogeny, it is laborious, time-consuming, costly, and subjective.

## Conclusion

It indicated that the incidence rate of *K*. *pneumoniae* in respiratory-diseased cats was high with a significant appearance of antibiotic resistance in study isolates. Sequencing data referred to that close related association of study isolates to human *K*. *pneumoniae* isolates, suggesting that nosocomial diseases were increased significantly in the veterinary field. Current identification and antibiotic susceptibility of *K*. *pneumoniae* could support the treatment protocol in respiratory-diseased cats since the appropriate selection can help in reducing the overall time of cure and reducing the associated risks and costs. Microbiological identification using different diagnostic techniques, in particular molecular assays, is recommended to obtain the greatest accuracy and to limit misidentification.

## List of abbreviations

BHI, Brain Heart Infusion

DNA, deoxyribonucleic acid

h, hour

NCBI, National Center For Biotechnology Information

PCR, polymerase chain reaction

RNA, ribonucleic acid

rRNA, ribosomal RNA

TSI, triple sugar iron agar

°C

degree celsius

μl, microliter.
